# Heterologous expression of *Pycnoporus cinnabarinus *cellobiose dehydrogenase in *Pichia pastoris *and involvement in saccharification processes

**DOI:** 10.1186/1475-2859-10-113

**Published:** 2011-12-28

**Authors:** Mathieu Bey, Jean-Guy Berrin, Laetitia Poidevin, Jean-Claude Sigoillot

**Affiliations:** 1 INRA, UMR1163 BCF, 163 avenue de Luminy, 13288 Marseille, France; 2 Aix-Marseille Universités, Faculté des Sciences de Luminy, 163 avenue de Luminy, 13288 Marseille, France; 3 IFP, Institut Français du Pétrole, 1 & 4 avenue de Bois-Préau 92852 Rueil-Malmaison, France

**Keywords:** White-rot fungi, CDH, gluconic acid, lignocellulose, biomass, saccharification

## Abstract

**Background:**

Cellobiose dehydrogenase (CDH) is an extracellular hemoflavoenzyme produced by lignocellulose-degrading fungi including *Pycnoporus cinnabarinus*. We investigated the cellulolytic system of *P. cinnabarinus*, focusing on the involvement of CDH in the deconstruction of lignocellulosic biomass.

**Results:**

First, *P. cinnabarinus *growth conditions were optimized for CDH production. Following growth under cellulolytic conditions, the main components secreted were cellulases, xylanases and CDH. To investigate the contribution of *P. cinnabarinus *secretome in saccharification processes, the *Trichoderma reesei *enzymatic cocktail was supplemented with the *P. cinnabarinus *secretome. A significant enhancement of the degradation of wheat straw was observed with (i) the production of a large amount of gluconic acid, (ii) increased hemicellulose degradation, and (iii) increased overall degradation of the lignocellulosic material. *P. cinnabarinus *CDH was heterologously expressed in *Pichia pastoris *to obtain large amounts of pure enzyme. In a bioreactor, the recombinant CDH (rCDH) expression level reached 7800 U/L. rCDH exhibited values of biochemical parameters similar to those of the natural enzyme, and was able to bind cellulose despite the absence of a carbohydrate-binding module (CBM). Following supplementation of purified rCDH to *T. reesei *enzymatic cocktail, formation of gluconic acid and increased hemicellulose degradation were observed, thus confirming the previous results observed with *P. cinnabarinus *secretome.

**Conclusions:**

We demonstrate that CDH offers an attractive tool for saccharification process enhancement due to gluconic acid production from raw lignocellulosic material.

## Background

In natural environments, cellulolytic microorganisms secrete enzymes that function synergistically, in association with the microorganism or independently. Although it is not fully known how many enzymes are involved in cell wall deconstruction, three general categories of enzymes are considered necessary to hydrolyze native cell wall materials: cellulases, hemicellulases and accessory enzymes such as hemicellulose debranching enzymes, phenolic acid esterase, and possibly lignin-degrading and modifying enzymes [1].

The main industrial source of cellulases and hemicellulases is the mesophilic soft-rot fungus *T. reesei *(teleomorph *Hypocrea jecorina*), valued for the high protein secretion capacity of its mutant strains obtained by random mutagenesis (producing up to 100 g of extracellular protein per liter of culture) [2,3].

Among fungal classes, basidiomycetes are known to be efficient degraders of cellulose, many species growing on dead wood or litter. The lignocellulolytic system of basidiomycetes has been studied intensively in the last decades. Genome sequencing and proteomic tools are often used, but the cellulolytic system is still not completely understood, especially the oxidative part of this system [4,5].

Cellobiose dehydrogenases (CDH; E.C. 1.1.99.18; cellobiose: [acceptor] 1-oxidoreductase) are extracellular fungal hemoflavoenzymes produced by many white-rot fungi including *Trametes versicolor, Phanerochaete chrysosporium, Ceriporiopsis subvermispora *and *P. cinnabarinus *[6-9]. CDH are also produced by the brown-rot fungus *Coniophora puteana *[10] and the soft-rot fungus *Humicola insolens *[11]. More recently, CDH from the ascomycetes *Myriococcum thermophilum *[12] and *Neurospora crassa *[13] were cloned and successfully expressed in *Pichia pastoris*. CDH are monomeric enzymes carrying two prosthetic groups, a heme and a flavin domain. The heme-binding domain in the N-terminal position contains a cytochrome b-type heme which presents an unusual heme binding by Met/His ligation [14]. The flavin domain in C-terminal binds FAD non-covalently and is classified as a member of the glucose-methanol-choline family of oxidoreductases. These two regions are separated by a Thr-Ser-rich long linker region [15]. The flavoprotein domain of CDH catalyzes two-electron oxidation of cellobiose and more generally cellodextrines, mannodextrines and lactose to corresponding lactones [16] using electron acceptors such as dioxygen, quinones, phenoxyradicals and others [17-19]. Also, one-electron transfer occurs. Heme is implicated in one internal electron transfer to FAD or another electron acceptor such as Fe^3+ ^[20,21].

Among oxidoreductases, laccases have been the most intensively studied, while CDHs are less well-researched. To date, thirteen CDHs have been characterised (*P. chrysosporium *[7], *Sclerotium *(*Athelia*) *rolfsii *[22], *Monilia sp*. [23], *T. versicolor *[9], *Trametes hirsuta *[24], *P. cinnabarinus *[25], *Schizophyllum commune *[26], *C. subvermispora *[6], *Sporotrichum thermophilum *[27], *C. puteana *[10], *Chaetomium sp*. [28], *M. thermophillum *[12], *Termitomyces clipeatus *[29], *H. insolens *[11], *Grifola frondosa *[30] and *N. crassa *[13]).

Although the role of CDHs is still unclear, it is established that CDHs are produced in cellulolytic conditions and are involved in cellulose and lignin degradation. CDHs have been shown to bind cellulose in different ways depending on species: a long aromatic-rich region for *P. chrysosporium *[31] or a cellulose-binding domain for ascomycetes and soft-rot fungi, similar to that observed for cellulases [32]. Their involvement in many reactions has been demonstrated, e.g. reduction of quinones [33,34], inhibition of phenol radical repolymerization [35,20], production of hydrogen peroxide [36,37] and one of the most often cited reactions, the production of hydroxyl radicals by a Fenton-type reaction, which may participate in the degradation of cellulose, lignin and xylan [38]. CDHs are known to enhance the action of cellulases on crystalline cellulose [39,40] and also to degrade wood components, but their role in complex lignocellulosic substrate degradation has never been investigated.

Here we examined the cellulolytic system of *P. cinnabarinus *and the involvement of CDH therein. Given its relevance to saccharification processes, we heterologously expressed the *P. cinnabarinus *CDH in *Pichia pastoris*. The recombinant enzyme was thoroughly characterized and assessed for its ability to degrade natural substrate as a supplement to commercial *Trichoderma reesei *cocktail.

## Results

### Production and characterization of *P. cinnabarinus *ss3 secretome in cellulolytic conditions

CDH is produced by *P. cinnabarinus *when cellulose is added to the culture medium. The best production (355 U/L) appeared after 10 days of cultivation when cellulose was used as sole carbon source. To understand the role of CDH when secreted in cellulolytic conditions, we characterized the *P. cinnabarinus *secretome after 11 days of growth.

Main enzymatic activities present in *P. cinnabarinus *secretome in cellulolytic conditions were measured by assay on a range of substrates (Table 1). No significant laccase or peroxidase activities were detected under our experimental conditions. However, *P. cinnabarinus *secretome contained enzymes able to hydrolyze a broad range of polysaccharides. Significant levels of activities towards pNP-glucose, CMC and pNP-cellobiose were detected, corresponding to β-glucosidase (0.35 U/mg), endoglucanase (0.55 U/mg) and cellobiohydrolase (0.32 U/mg). A variety of hemicellulases were also identified in *P. cinnabarinus *secretome. The two main endo-glycosidase activities were present corresponding to endo-mannanase and endo-xylanase with about 2 U/mg. Hemicellulase exoglycosidase enzymes were detected to a lesser extent: 0.85 U/mg of α-galactosidase and 0.01 U/mg of β-xylosidase were measured.

**Table 1:** Lignocellulose-degrading enzyme activities measured in *P.cinnabarinus *secretome

Type of activity	Substrate	Activity (U/mg)
CDH	Cellobiose	0.53
Laccase	ABTS	nd^a^
Glucose oxidase	Glucose	nd^a^
Manganese peroxidase	Vanillyl acetone	nd^a^
Lignin peroxidase	Veratryl alcohol	nd^a^
β-Glucosidase	pNP-glucose	0.35 ± 0.00
Endoglucanase	CMC	0.55 ± 0.00
Cellobiohydrolase	pNP-Cellobiose	0.32 ± 0.00
β-Xylosidase	pNP-Xylose	0.01 ± 0.00
Endo-xylanase	Low viscosity arabinoxylan	2.03 ± 0.08
β-Mannosidase	pNP-Mannose	nd^a^
Endo-mannanase	Galactomannan	2.03 ± 0.11
Pectinase	Pectin	0.45 ± 0.02
α-Galactosidase	pNP-Galactose	0.85 ± 0.00

Results are expressed in U/mg of total proteins.

^a ^no activity detected

Zymogram assays were performed on the culture extract of *P. cinnabarinus *to give insight into the number of isoforms present for the main enzymatic activities previously measured.

**Figure 1:**
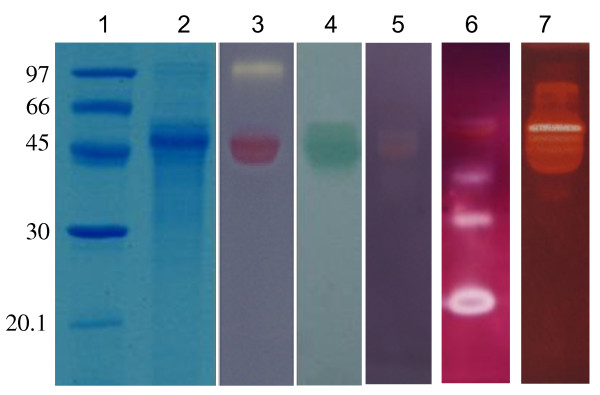
**SDS-PAGE and zymogram on the supernatant of *P. cinnabarinus *ss3 grown in cellulolytic conditions**. 1, pre-stained molecular weight marker; 2, SDS-PAGE with 10 μg of protein supernatant after ultrafiltration step; 3, CDH zymogram; 4, zymogram with ABTS; 5, soluble xylan birchwood (xylanase) zymogram; 6, CMC (endoglucanase) zymogram and 7, locust bean gum (mannanase) zymogram.

SDS-PAGE of *P. cinnabarinus *(Figure 1, lane 2) grown in cellulolytic conditions presented two main differences when compared with reference culture supernatant grown in non-cellulolytic conditions: (i) the presence of a band around 100 kDa, attributable to CDH and (ii) the absence of 70 kDa band corresponding to laccase. Confirmation by the zymogram technique showed the DCPIP decoloration by the 100 kDa band corresponding to CDH activity (Figure 1, lane 3). Oxidation of ABTS occurred at around 50 kDa (Figure 1, lane 4) and could not be attributed to laccase, which has a molecular mass of 70 kDa. The xylanase zymogram (Figure 1, lane 5) demonstrated the presence of a weak activity at 50 kDa corresponding to the results previously described [41]. CMCase zymogram (Figure 1, lane 6) showed at least five bands with the brightest one at 25 kDa. For mannanase activity (Figure 1, lane 7), some bands were represented around one major band at 60 kDa.

### *P. cinnabarinus *CDH *sequence *analysis

Based on *P. cinnabarinus *ss3 *cdh *sequence, primers were designed to clone the *cdh *gene starting from 4-day-old culture induced with cellulose. The *cdh *sequence of 2310 bp was compared with available *cdh *sequences. Nucleotide sequence analysis showed 97% identity between *cdh *of *P. cinnabarinus *I-937 described by Moukha *et al*. [8] and the *cdh *from *P. cinnabarinus *ss3, a monokaryotic strain isolated from the fruit-like structure of *P. cinnabarinus *I-937, a wild-type dikaryotic strain. These observed differences in the nucleotide sequence resulted in eight amino acid differences at positions 96 (Ala→Glu), 331 (Arg→Ser), 354 (Ala→Thr), 357 (Asn→Lys), 386 (Tyr→Ser), 426 (Tyr→Phe) and 495 (Gln→Glu). Comparison with *T. versicolor *CDH and *P. chrysosporium *CDH resulted in amino acid sequence identities of 77% and 70%, respectively. *P. cinnabarinus *CDH amino acid sequence exhibited conserved regions with GMC oxidoreductase [42] conserved domain. The linker region rich in Thr-Ser (from position 182 to position 215), the FAD binding site and the Met/His ligands for heme fixation were also identified. Interestingly, the Thr-Ser region was also rich in Pro (28% Thr, 25% Pro, 13% Ser). Analysis of the gene encoding the CDH from *P. cinnabarinus *has shown high sequence homology with *cdh *from class I. Indeed, phylogenetic analysis of *cdh *genes revealed two major classes [42]. The class I *cdh *genes are found only in basidiomycetes while the class II contain more complex ascomycetes CDHs, that sometimes present a family 1 carbohydrate-binding module (CBM) at the C-terminal position. Emergence of a third class of CDHs in ascomycetes fungi was recently reported [32].

### Heterologous expression of CDH in *P. pastoris*

The coding sequence of *cdh *was inserted into the *P. pastoris *expression vector in frame with sequences encoding the yeast α-factor secretion peptide and a (His)_6 _tag located at the C terminus. The recombinant gene was then introduced into the *Pichia *genome under the control of the methanol-inducible promoter. Multi-copy transformants were screened to select a clone that exhibited satisfactory levels of production. CDH activity was successfully detected in the supernatant after induction, indicating correct processing of the α-factor signal sequence.

A maximum activity of 1176 U/L was observed after 4 days of induction, and this clone was chosen for this study. To scale up enzyme production, we optimized CDH expression in a 1 liter bioreactor with the best-performing clone of *P. pastoris*. The recombinant CDH was secreted at high levels, reaching 7800 U/L. Recombinant CDH was purified after 4 days of induction, taking advantage of the (His)_6 _tag. Also, only trace amounts of endogenous proteins were present in the culture supernatant of the transformant secreting CDH. The purified enzyme exhibited a specific activity of 22.2 U/mg.

### Biochemical characterization of rCDH

**Figure 2:**
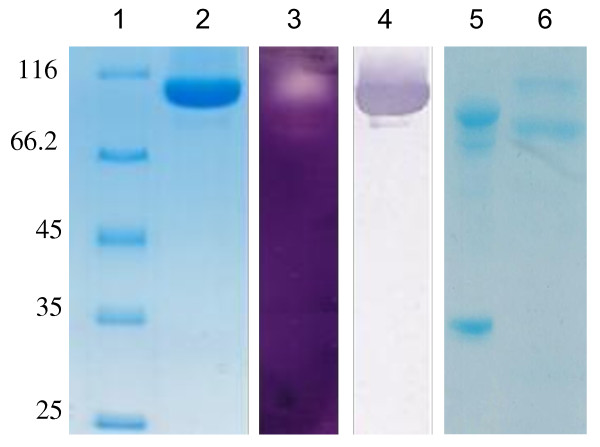
**SDS-PAGE, zymogram, immunoblot analysis, deglycosylation and cleavage by papain of purified recombinant rCDH**. 1, pre-stained molecular weight marker; 2, 10 μg of purified CDH; 3, CDH zymogram on the sample 2; 4, Western blot analysis using anti-His antibodies; 5, CDH deglycosylated; 6, CDH digested by papain.

Recombinant CDH was purified to homogeneity, i.e. one major band displaying a relative molecular weight around 110 kDa (Figure 2, lane 2) appeared on SDS-PAGE. Western blot analysis (Figure 2, lane 4) confirmed the presence of CDH at 110 kDa.

Zymogram analysis of CDH activity revealed active bands on the gel corresponding to 70 and 110 kDa (Figure 2, lane 3). Deglycosylation (Figure 2, lane 5) of CDH showed enhanced degradation between the two enzyme moieties and a loss of approximately 10 kDa. Following papain cleavage of CDH, a band corresponding to the FAD-containing moieties was observed on SDS-PAGE (Figure 2, lane 6). The heme-containing domain was not seen after staining, probably owing to the weak presence of aromatic residues [43].

**Figure 3:**
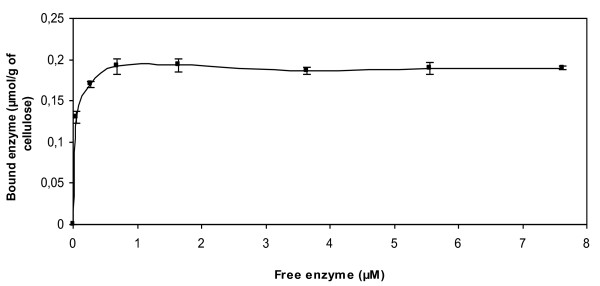
**Kinetics of the binding of rCDH**. Experiments were performed on Avicel at 30°C, pH 4.5. Error bars indicate standard deviations from triplicate measures performed independently.

Binding studies of CDH confirmed the ability of the enzyme to bind cellulose without the presence of a cellulose-binding domain. Dissociation constant (*K_d_*) and binding capacities (*B_max_*) of CDH were determined and were respectively 0.064 μM and 0.2 μmol/g of Avicel (Figure 3).

**Figure 4:**
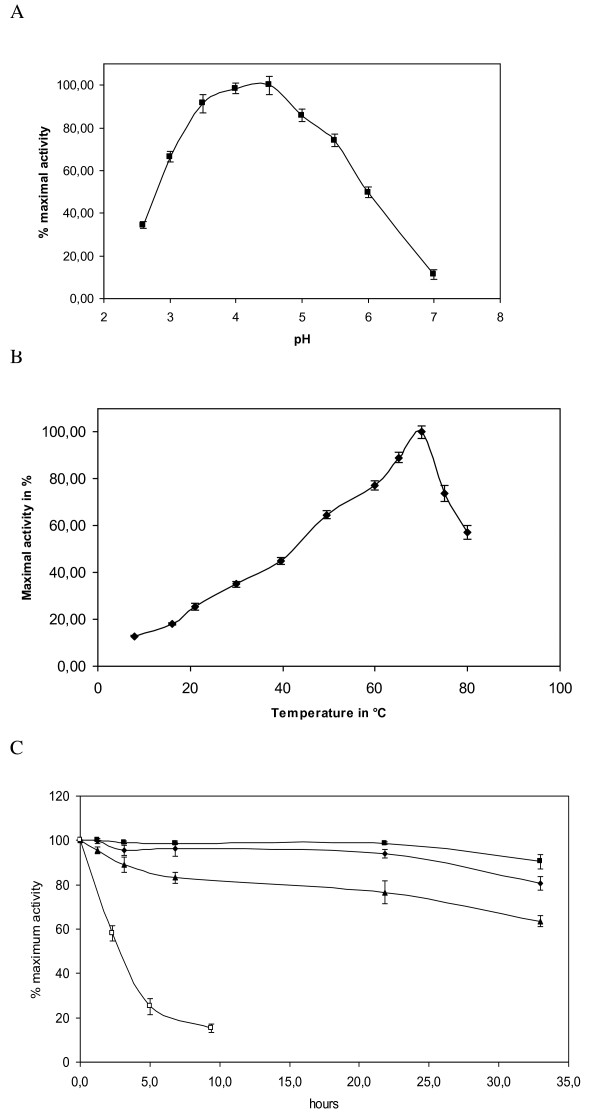
**Effect of temperature on the activity of purified rCDH using DCPIP as electron acceptor**. Activity was determined at pH 4.5 except for determination of optimal pH. (A) Optimal pH of purified rCDH using DCPIP as electron acceptor. Activity was determined at 30°C in citrate-phosphate buffer pH 2.7-7. (B) Temperature optimum. (C) Temperature stability at 45°C(■), 50°C(♦), 55°C(▲) and 65°C(□). Measured were performed at least three times.

When DCPIP is used as electron acceptor, the optimal temperature for CDH is 70°C. The recombinant enzyme displayed activity over a wide range of temperatures, 16% of residual activity at 10°C and 55% of residual activity at 80°C (Figure 4A).

After incubation of CDH at 45, 50 and 55°C for 33 h, residual enzyme activity was 90%, 80% and 63% respectively. However, CDH was not stable at 65°C, with only 15% of activity remaining after 9 h (Figure 4C). The optimal pH for recombinant CDH (Figure 4A) was pH 4.5. The recombinant CDH had *V_max _*= 22.2 U/mg and *K_M _*= 35.5 μM for cellobiose with DCPIP as electron acceptor. Using cyt c as electron acceptor, we found *V_max _*= 3.9 U/mg and *K_M _*= 14.7 μM (Table 2).

**Table 2:** Apparent kinetic constants of purified rCDH for cellobiose

Electron acceptor	*K*M (μM)	*V*max ^a ^(μmol/min/mg)	*k*cat (s-1)	*k*cat/*K*M (s-1. mM-1)
DCPIP	35.5	22.2	40.8	1148
Cyt c	14.7	3.9	7.0	474

Reactions were performed at 30°C, pH 4.5 using selected electron acceptors.

^a ^Kinetic parameters were determined using DCPIP or cyt c as electron acceptor and cellobiose as substrate under standard assay conditions.

### Effect of CDH on the saccharification of wheat straw

The range of lignocellulosic enzymes found in the supernatant of *P. cinnabarinus *makes it a candidate for supplementation of the *T. reesei *cocktail for saccharification of wheat straw. We thus set out to compare the efficiency of the *P. cinnabarinus *supernatant with the purified rCDH for supplementation of industrial cocktails.

**Figure 5:**
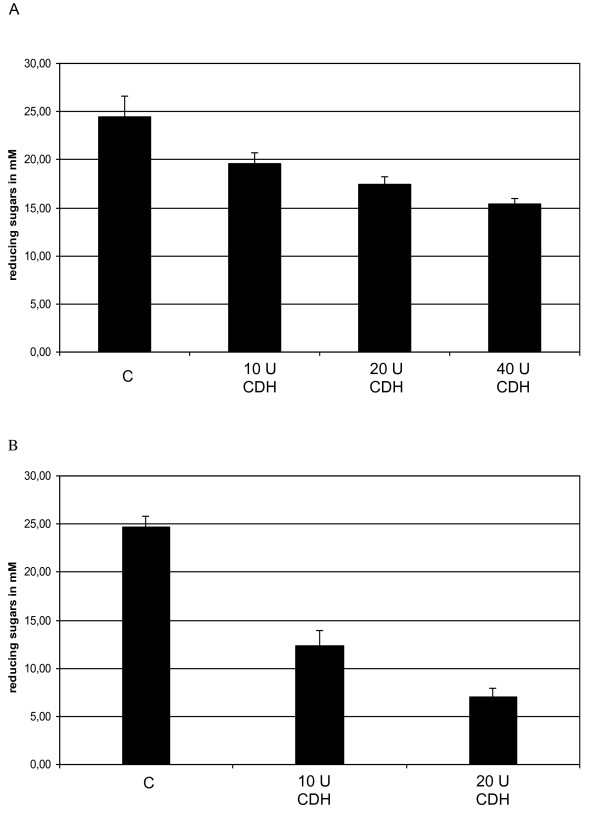
**Analysis of total reducing sugar yield after 96 h of enzymatic treatment on wheat straw**. Reducing sugars (mM) release during hydrolysis were quantified spectrophotometrically using the DNS method as described in Navarro *et al*., 2010 [69]. Assay conditions for hydrolysis were conducted in 20 mL of 50 mM sodium phosphate buffer (pH 4.8) containing 5% (w/v) wheat straw. (A) *P. cinnabarinus *secretome containing CDH or (B) purified rCDH were assayed in the presence of cellulose cocktail at 45°C with an orbital agitation (140 rpm) during 96 h. C: control reaction without CDH. Error bars are for triplicate, each measured once.

**Figure 6:**
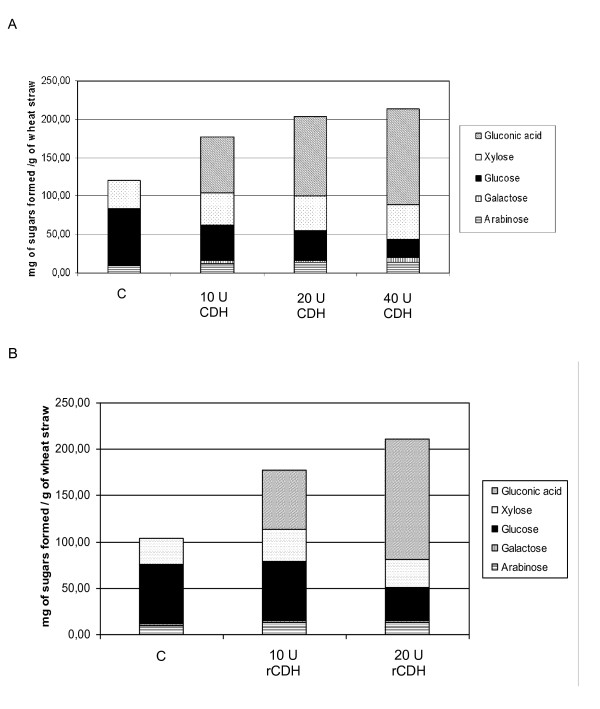
**Determination of sugar yield in mg of sugars formed/g of wheat straw after 96 h of enzymatic treatment on wheat straw**. Saccharification assays were performed in 20 mL of 50 mM sodium phosphate buffer (pH 4.8) containing 5% (w/v) wheat straw. (A) *P. cinnabarinus *secretome containing CDH or (B) purified rCDH were assayed in the presence of cellulose cocktail at 45°C with an orbital agitation (140 rpm) during 96 h. C: control reaction without CDH. Sugars were quantified by high-performance anion exchange chromatography (HPAEC) coupled with amperometric detection (PAD) equipped with a Carbo-Pac PA-1 analytical column. These experiments were repeated at least three times with similar results. For all-sugars analysis in (A) and (B) statistical significance was *P *< 0.1 and *P *< 0.05 respectively.

The *T. reesei *cocktail supplemented with β-glucosidase was used as reference. Addition of *P. cinnabarinus *secretome (10, 20 and 40 U of CDH) or pure rCDH (10 and 20 U) gave similar results (Figure 5 and Figure 6). DNS assays were used to measure reducing ends of sugars released after saccharification. Supplementation with purified rCDH or secretome containing CDH showed less response on DNS titration than control with cocktails (Figure 5). However, overall hydrolysis was increased by addition of CDH, with the production of large amounts of gluconic acid, from 5 to 100 mg per g of wheat straw, compared with control. Also, greater yields of xylose, galactose and arabinose, which increased respectively from 35 to 44, 1.9 to 4 and 9.5 to 13.5 mg/g of wheat straw were observed with addition of 10 U of CDH (Figure 6A). Production of gluconic acid by CDH can be explained by the formation of cellobionolactone following by its spontaneous hydrolysis in cellobionic acid. This last compound can be cleaved by β-glucosidase into glucose and gluconic acid. Experiments were performed on Dionex (data not shown).

To confirm the strong production of gluconic acid, purified recombinant CDH was used to supplement the *T. reesei *and *A. niger *cocktails. The effect on wheat straw was comparable to that obtained with *P. cinnabarinus *secretome (Figure 6B). Also, supplementation with 10 U of rCDH did not affect the yield of glucose, increased hemicellulose yield and resulted in the formation of gluconic acid in large amounts.

## Discussion

In the last few decades the white-rot fungus *P. cinnabarinus *has been studied for its ligninolytic system, which is based on phenoloxidases such as laccases, without the presence of peroxidases [44]. This system, and especially laccase, has been used to produce high value compounds [45,46] and applied to the design of biotechnological processes [47]. Here we investigated the cellulolytic and oxidative system of *P. cinnabarinus *grown in cellulolytic conditions.

In the *P. cinnabarinus *secretome, we found hemicellulase activities already reported in the literature: α-galactosidase, xylanase or β-galactosidase [48,49,41], together with mannosidase and arabinofuranosidase activities not hitherto described in *P. cinnabarinus*. Endoglucanase and exoglucanase were identified by zymogram (CMCase) and by hydrolysis of Avicel and CMC. Peroxidase activity assay (manganese peroxidase and lignin peroxidase) was performed on the secretome, but no activity was recovered. *P. cinnabarinus *is a well-known producer of laccase [50], but in cellulolytic conditions, laccase production seems to be repressed, whereas the zymogram shows activity on ABTS around 50 kDa. Similar results were observed in *P. chrysosporium *grown in cellulolytic condition with the presence of several laccase bands on the zymogram around 50 kDa confirmed by electron paramagnetic resonance [51]. Production of CDH was previously described [41,25] and its activity was followed in *P. cinnabarinus *culture.

We cloned and expressed *P. cinnabarinus *CDH in *P. pastoris*. CDH of *T. versicolor *[52], *P. chrysosporium *[53] and more recently *N. crassa *[13] were previously expressed in the same host. These results confirm that *P. pastoris *heterologous expression is an efficient way to produce fungal CDHs at high levels.

Enzymatic characterization of recombinant CDH gave values of kinetic parameters (*V*_max_, *K*_M_) in the same range as those observed previously for the native enzyme [25] and more generally for the recombinant CDH cited in the literature [12,52]. However, recombinant CDH of *P. cinnabarinus *is more thermostable than the other fungal CDHs, with an optimal temperature around 70°C. Optimal pH 4.5 is in close agreement with the literature.

Some CDHs produced by ascomycetes and soft-rot fungi contain a carbohydrate binding module (CBM) and are able to bind cellulose. In the case of *P. chrysosporium *CDH, the ability to bind cellulose seems to be mediated by a specific domain with a structure different from CBM [31]. The ability of the purified enzyme to bind Avicel in the absence of CBM was confirmed experimentally.

CDH is produced simultaneously with cellulase. Its role in the degradation of cellulose was shown by Bao *et al*., who found that *P. chrysosporium *CDH increased the sugar yield from cellulose and produced cellobionolactone [39]. In this work, we decided to use CDH to supplement cellulase cocktail on complex substrate such as wheat straw.

In a first set of experiments, we used the *P. cinnabarinus *secretome containing CDH added directly to cellulase cocktail for the saccharification of wheat straw.

Results on wheat straw showed (i) increased yield in C5 sugars from hemicelluloses, consistent with the lignin degradation effect of the secretome, and (ii) a slight decrease in glucose yield correlated with the formation of large amounts of gluconic acid due to cleavage of cellobionic acid (the main product of the reaction performed by CDH) by β-glucosidase.

Supplementation with purified rCDH gave similar results on wheat straw and even no decrease in glucose yield, but gluconic acid and C5 sugar hemicellulose production was enhanced for 10 U CDH supplementation. Results point to synergy between CDH and cellulases for degradation of raw material. In *P. cinnabarinus *secretome, β-glucosidase activity was significantly detected (Table 1). However, when no β-glucosidase was added to the saccharification assay, more cellobionic acid was produced instead of gluconic acid by *T. reesei *cocktail supplemented with *P. cinnabarinus *secretome (data not shown). It is well established that β-glucosidases are inhibited by gluconolactone and more generally that lactones are inhibitors of many glycosidases [54,55]. Nevertheless, sugar lactones are unstable in aqueous solution, and the rate of spontaneous hydrolysis to the corresponding aldonic acid, i.e. gluconic acid or cellobionic acid, depends on the pH and temperature of the reaction. Aldonolactonase, found in several fungi, catalyzes the hydrolysis of lactones to aldonic acid [56]. This hydrolysis should relieve inhibition of β-glucosidase and glycosidase by lactone, as suggested by Bruchman *et al*. [57]. β-Glucosidase is able to cleave cellobionic acid into glucose and gluconic acid [58]; cellobionic acid and gluconic acid production decreases the number of reducing ends as shown by the decrease in DNS titration. In the presence of CDH, DNS titration is not a relevant method for monitoring cellulose degradation. The presence of cellobionic acid seems due to a faster reaction rate of CDH than β-glucosidase *versus *cellobiose, as shown by Yoshida *et al*. [59]. Supplementation with β-glucosidase compensates for the difference in reaction rate, leading to a greater production of gluconic acid. Conversely, as the accumulation of cellobiose induces inhibitory effects on cellulase [60], CDH may decrease the cellobiose concentration in the medium faster and so avert inhibition.

## Conclusions

Supplementation of *T. reesei *secretome by CDH increases the overall degradation of lignocellulose and produces appreciable amounts of gluconic acid. In saccharification processes, the use of gluconic acid should offer a way to improve the profitability of the whole process. Several organisms use gluconic acid through the pentose phosphate pathway. *Zymomonas mobilis*, for example, is able to produce ethanol from gluconic acid by the Entner-Doudoroff pathway [61]. Alcoholic fermentation from gluconic acid by *Saccharomyces bulderi *has also been reported [62]. The introduction of such organisms able to use pentose and gluconic acid should increase the overall yield of ethanol by using less fermentable components and should offer a way to design a sustainable process for second generation bioethanol production.

## Methods

### Biological material

*P. cinnabarinus *ss3 monokaryotic strain BRFM 137 isolated from the fruit-like structure of the *P. cinnabarinus *I-937 dikaryotic strain was maintained as previously described [50]. *P. pastoris *strain X33 is a component of the *Pichia *Easy Select Expression System and the pPICZαA vector (Invitrogen, Cergy-Pontoise, France).

### Media and culture conditions

*P. cinnabarinus *was grown at 30°C on MYA2 plates (maltose: 20 g/L; yeast extract: 1 g/L; agar 16 g/L). After 10 days of incubation, precultures in Roux flasks containing 200 mL of medium were inoculated with five disks of *P. cinnabarinus *grown in MYA_2 _plates. Inoculum was obtained from 10-day-old static precultures incubated at 30°C.

We used 10 mL of inoculum suspension obtained from Ultra-Turrax-mixed mycelial mats to inoculate 500 mL baffled conical flasks containing 250 mL of basal medium composed of cellulose fibrous medium (Sigma, St. Louis, Mo, USA) (15 g/L), diammonium tartrate (1.84 g/L), disodium tartrate (2.3 g/L), KH_2_PO_4 _(1.33 g/L), CaCl_2_.H_2_O (0.1 g/L), MgSO_4_.7H_2_O (0.5 g/L), FeSO_4_.7H_2_O (0.07 g/L), ZnSO_4_.7H_2_O (0.046 g/L), MnSO_4_.H_2_O (0.035 g/L), CuSO_4_.5H_2_O (0.007 g/L), yeast extract (1 g/L), vitamin solution (1 mL/L) according to Tatum *et al*. [63] maltose (2.5 g/L) used as starter and Tween 80 (1.5 g/L), according to [41]. For the heterologous expression of CDH in *Pichia pastoris*, all media and protocols are described in the *Pichia *expression manual (Invitrogen). Cloning procedures were performed using one-shot TOP 10 and DH5α chemically competent *Escherichia coli *cells (Invitrogen).

### Isolation of mRNA and cloning of *cdh *cDNA gene

Isolation of total RNA was performed on a 4-day-old culture of *P. cinnabarinus *on cellulose medium using Total RNA Purification from Plant (Macherey-Nagel, Düren, Germany) as prescribed by the manufacturer. Contaminant DNA was digested by Turbo DNase (Ambion Inc., Austin, TX, USA) according to the manufacturer's instructions. First-strand cDNA synthesis was performed using SuperScript reverse transcriptase (Invitrogen) and oligo(dT_18_) primer following the manufacturer's instructions. The amplification of the full-length *cdh *cDNA was performed using specific primers (with restriction sites underlined): forward primer cdhF (5' TA GAA TTC CAA GTG GCA GCG CCA TAC 3') and reverse primer cdhR (5' TA TCT AGA CCA GGA CCT CCC GCA AGG GC 3') designed from *P. cinnabarinus *I-937 *cdh *gene (NCBI AF081574): 315 ng of cDNA was mixed with 300 pmol of each primer cdhF and cdhR, 200 μM dNTPs, and 0.5U *Pfu *DNA polymerase (Promega, Madison, WI, USA). The reaction was performed with the following amplification program: 1 cycle at 95°C for 5 min, 30 cycles composed of three steps for each cycle (95°C for 1 min, 65°C for 30 s and 72°C for 4 min), and a final step at 72°C for 10 min. PCR amplicons generated by *Pfu *DNA polymerase are blunt-ended. To add an A-tail on these PCR fragments before subcloning into pGEMT-easy vector, *Taq *DNA polymerase (Promega) was used as described in the pGEMT-easy vector Technical Manual (Promega). The 2.3 kb PCR product was purified using the Qiaquick gel extraction kit (Qiagen, Valencia, CA, USA) and subcloned into pGEMT easy vector.

The *cdh *cDNA was further sequenced (GATC Biotech, Mulhouse, France) using sp6 and T7 universal primers and cdhint (5' CGA CGC CCA GAA CTC GAA C 3'). The *P. cinnabarinus *sequence was deposited in the NCBI databank (GenBank accession number: BankIt1421219 Pycnoporus HQ825322). Comparisons of *P. cinnabarinus *I-937 *cdh *(GenBank accession number: AF081574.1), *T. versicolor cdh *(GenBank accession number: AY187939.1) and *P. chrysosporium cdh *(GenBank accession number: U46081.1) were performed with ClustalW2 software http://www.ebi.ac.uk/Tools/msa/clustalw2/.

### Construction of pPiCZαA expression vector

The *cdh *cDNA cloned into pGEMT easy vector was digested using *Eco*RI and *Xba*I and purified with a Qiaquick gel extraction kit. In parallel, pPICZαA was linearized using the same restriction enzymes, and *cdh *cDNA was ligated at the corresponding sites into pPICZαA in frame with both the yeast α-secretion factor and C-term-(His)_6_-tag encoding sequences. Expression vector pPICZαA-*cdh *was purified by Qiagen Midiprep and sequenced using 3'AOX and 5'AOX primers to confirm the correct sequence insertion.

### Transformation and screening

Transformation of competent *P. pastoris *X33 was performed by electroporation with *Pme*I linearized pPICZα A-*cdh *as described in Couturier *et al*. 2010 [64]. The vector pPICZα without insert was used as a control. Transformants were first screened on YPDS plates with different concentrations of zeocin (100 to 1000 μg/mL). After incubation at 30°C, transformants were picked from minimal dextrose (MD) plates and transferred to minimal methanol plates (MM). Zeocin-resistant *P. pastoris *transformants were then screened for protein expression in 10 mL of BMGY (in 50 mL tubes) at 30°C in an orbital shaker (200 rpm) for 16 h to an OD_600 _of 2-6, and expression was induced by transferring cells into 2 mL of BMMY and growing for a further 3 days. Each day the medium was supplemented with 3% (v/v) methanol. The supernatant was then analyzed by SDS-PAGE to determine which transformant had the best secretion yield.

### Recombinant CDH production

The best-producing transformant was grown in 1 liter of BMGY in shaken flasks as described above. The cells were then transferred to 200 mL of BMMY and stirred at 200 rpm at 30°C for 4 days.

Bioreactor production of the best-producing transformant was carried out in a 1-liter bioreactor Tryton (Pierre Guerin, Mauze, France) according to the Pichia Fermentation Process Guidelines (Invitrogen) except for the volume of methanol added in the methanol fed batch, which was changed from 3.6 mL/h/L to 3 mL/h/L.

### Enzyme purification

Culture supernatant was concentrated at least 10 times using Amicon centrifugal units with a 30 kDa cut-off, 4000× *g *or Amicon vivaflow (Millipore, Bedford, MA, USA) with a 30 kDa cut-off, depending on culture volume. The concentrated supernatant was dialyzed against buffer A (Tris-HCl 50 mM 7.8, NaCl 150 mM and imidazole 10 mM), and loaded on a nickel chelate His-Bind Resin (GE Healthcare, Buc, France) column (0.7 × 5 cm) connected to an Äkta FPLC (GE Healthcare) and equilibrated with buffer A. The His-tagged rCDH was eluted with buffer B (Tris-HCl 50 mM pH 7.7, imidazole 500 mM and NaCl 150 mM). Active fractions were pooled, concentrated and dialyzed against sodium acetate buffer (50 mM, pH 5)

### SDS-PAGE, Western blot and zymogram

Polyacrylamide gel electrophoresis (SDS-PAGE) (12%) was prepared as described by Laemmli [65]. Protein bands were stained with Coomassie blue G 250. The molecular mass under denaturating conditions was determined with reference standard proteins (LMW, Amersham Pharmacia Biotech, Orsay, France or unstained protein molecular weight marker, Euromedex, Souffelweyersheim, France).

Enzyme activities were assayed in polyacrylamide gels containing the appropriate substrates. Enzyme preparations were run on an SDS-PAGE gel copolymerized with 0.2% soluble xylan, 0.2% carboxymethylcellulose (CMC) or 0.2% locust bean gum for the analysis of xylanase, CMCase or mannanase activities, respectively. The protein samples were mixed in the loading buffer (3% SDS w/v, 10% glycerol w/v and 30 mM Tris-HCl buffer pH 6.8) without reducing agent, heated at 100°C for 1 min and then separated using a 12% polyacrylamide gel. After electrophoresis, the gel was washed with de-ionized water and soaked in 2.5% (v/v) Triton X-100. After 1 h incubation at 4°C, the gel was soaked in 100 mM sodium phosphate buffer (pH 5) at 45°C for 2 h for the detection of xylanase and CMCase activity or in 100 mM sodium phosphate buffer (pH 7) for 1 h at 50°C for the detection of mannanase activity. After incubation the gel was stained with 0.1% Congo red solution under gentle shaking for 1 h and destained with 1 M NaCl for 1 h. Protein bands exhibiting xylanase, CMCase and mannanase activity were seen as clear bands on the red background.

For laccase and CDH zymograms, samples were mixed with the same loading buffer as described above without heating; they were incubated at ambient temperature for 15 min and the gel was run. After electrophoresis the gel was soaked in 2.5% Triton X-100 for 1 h at 4°C, rinsed with deionized water and incubated for 2 h at 25°C in 50 mM sodium acetate buffer (pH 5) with 4 mM sodium fluoride for CDH and 50 mM sodium tartrate buffer (pH 4) for laccase. Visualization was performed by adding 5 mM ABTS to stain for laccase and adding 50 mM DCPIP for CDH, staining the gel dark blue. CDH activity was then visualized by adding 100 mM cellobiose. Protein bands exhibiting CDH activity were seen as clear bands on the dark blue background. Western blot analysis was performed as described previously, using the monoclonal anti-polyhistidine alkaline phosphatase conjugate (Sigma) for Western blot analysis of rCDH expressed in *P. pastoris*. For Western blot analysis, purified rCDH was run on a 12% SDS/polyacrylamide gel and blotted onto a PVDF membrane using the iBlot Dry Blotting System (Invitrogen). Membranes were placed in a Snap Protein Detection System (Millipore, Bedford, MA, USA) used for immunodetection. Following the manufacturer's instructions, the PVDF membrane was incubated in TBS blocking solution (10 mM Tris, 150 mM NaCl and 0.1% Tween 20, pH 8) with addition of 0.1% (w/v) of skimmed milk powder and then washed with TBS. Immunodetection was performed using the monoclonal anti-polyhistidine alkaline phosphatase conjugate (Sigma,). Signal detection was carried out using 60 μL of BCIP (5-bromo-4-chloro-3-indolyl-phosphate), 60 μL of NBT (4-nitro blue tetrazolium) (Roche Applied Science, Meylan, France) in 20 mL carbonate buffer 0.05 M pH 9.6 with addition of 5 mM MgCl_2_.

Papain cleavage of the two CDH domains was carried out as described by Henriksson *et al*. [43]. Deglycosylation was performed using PGNase (New England Biolabs, Saint-Quentin-en-Yvelines, France) to remove rCDH N-linked glycans according to the manufacturer's instructions.

### Protein assay

Protein concentration was determined using the Bio-Rad Protein Assay (Bio-Rad, Marnes-la-Coquette, France), based on the Bradford procedure, using bovine serum albumin as standard [66].

### Binding studies

Assays were performed with 1 mg/mL of Avicel PH-101 (Sigma) in 50 mM citrate phosphate buffer pH 5 under orbital agitation at room temperature, and rCDH was added in the range 0.02-0.8 μg/L. After 3 h, Avicel PH-101 was removed by centrifuging, and the concentration of free enzyme ([FE], μM) in the supernatant was measured by activity assay. The bound enzyme concentration ([BE] in μmol per gram of Avicel PH-101) was determined by subtracting [FE] from the total protein concentration. All the assays were carried out in triplicate. Adsorption parameters were based on typical double-reciprocal plots using the equation [B] = [UB] × [B]_max_/ (*K*_d _+ [UB]), where *K*_d _(μM) and [B]_max _(μmol per gram of Avicel PH-101) are the equilibrium dissociation constant and the maximum amount of protein bound. Two controls were run, one without rCDH and the other with BSA at 1 μg/μL to estimate unspecific fixation of rCDH. Measures were repeated at least three times.

### Enzyme assays

To measure enzyme activities in the *P. cinnabarinus *culture supernatant, each aliquot was centrifuged for 5 min at 3500 rpm and filtered through a 0.45 μm membrane (Millipore, Bedford, MA, USA). CDH activities were determined by monitoring the reduction of 0.2 mM 2,6-dichlorophenol indophenol (DCPIP) in 100 mM sodium acetate buffer (pH 5) containing 2 mM cellobiose and 4 mM of sodium fluoride (sodium fluoride was used as a laccase inhibitor). The decrease in absorption at 520 nm (*ε *= 6800 M^-1^.cm^-1^) was monitored at 30°C for 1 min. Alternatively, CDH activity was determined by monitoring the reduction of 50 μM cytochrome c (cyt c) in 100 mM sodium-acetate buffer (pH 5) containing 2 mM cellobiose. The decrease in absorption at 550 nm (*ε *= 33,700 M^-1^.cm^-1^) was monitored at 30°C for 1 min. Glucose oxidase was measured using the D-gluconic acid / D-glucono-δ-lactone assay (Megazyme). Laccase activity was determined quantitatively by monitoring the oxidation of 5 mM ABTS (2, 2'-azino-bis (3-ethylbenzthiazoline-6-sulfonic acid)) at 420 nm (extinction coefficient 36,000 mM.^-1 ^cm.^-1^) in the presence of 50 mM NaK tartrate, pH 4.0. Lignin peroxidase activity was determined spectrophotometrically at 30°C by the method of Tien and Kirk [67]. Manganese peroxidase activity was determined spectrophotometrically at 30°C by the method of Paszczynski *et al*. [68] using H_2_O_2 _and vanillylacetone as substrate. Enzyme activity was expressed in international units (IU). One unit of activity is defined as the quantity of enzyme that transforms 1 μmol of substrate in one minute.

Hydrolysis assays for glycosidases were carried out in 50 mM acetate buffer pH 5 containing 1 mM of substrate in a final volume of 100 μL. Substrates pNP-β-D-glucopyranoside, pNP-β-D-cellobiopyranoside, pNP-β-D-xylopyranoside, α-D-galactopyranoside and pNP-β-D-mannopyranoside were purchased from Sigma. Assays were performed with 0.5 and 1 μg of protein, and incubated for 37°C for 1 h with shaking (300 rpm). To stop the reaction, 130 μL of Na_2_CO_3 _1 M was added, and absorbance was read at 410 nm. A control was run with 100 μL of 50 mM acetate buffer pH 5 and references ranging from 0.02 to 0.2 mM of 4-nitrophenyl were measured in parallel. Enzymatic activity was based on colorimetric assay of free pNP present in the reaction after hydrolysis. This activity is expressed in U/mg of proteins.

Hydrolysis assays were carried out in 50 mM acetate buffer pH 5 containing 1% (w/v) of substrates. Carboxymethyl cellulose (CMC, low viscosity) and citrus pectin were purchased from Sigma. Wheat arabinoxylan (low viscosity) and galactomannan (low viscosity) were from Megazyme. Assays were performed with 10 and 30 μg of protein, and incubated at 37°C for 1 h with shaking (150 rpm). Reducing sugars released during hydrolysis were quantified by DNS (3, 5-dinitrosalycylic acid) visualization at 540 nm as described in Navarro *et al*., 2010 [69]. Controls were run with 50 mM acetate buffer pH 5 and references ranging from 1 to 10 mM of glucose were measured in parallel for each series. Enzymatic activity is expressed in U/mg of proteins. Three controls were performed with the secretome alone to quantify sugars present in culture supernatant. Controls were subtracted from measured values. All assays were performed in triplicate.

### Effect of pH and temperature on the activity and stability of rCDH

To determine the optimum pH of the rCDH, the activity was measured with DCPIP using 50 mM citrate phosphate buffer in the pH range 2.5-7 at 30°C. For optimum temperature determination, activity on DCPIP was measured using 50 mM citrate phosphate buffer in the temperature range 10-80°C. Thermal stability of rCDH was determined by incubating enzymes for 33 h at 45, 50 and 55°C and for 10 h at 65°C. Native CDH activity assay was performed in triplicate as described above.

### Enzyme kinetics

The kinetic parameters (*V*_max _and *K*_m_) were determined for cellobiose oxidation measured at 30°C in 50 mM citrate phosphate buffer pH 4.5 using DCPIP or cytochrome c. The concentration of cellobiose ranged from 10 to 700 μM with both electron acceptors (DCPIP and cytochrome c). Triplicates were run to ensure reliable kinetic parameter determination.

Graphpad prism v.4 (Graphpad Software) was used for the nonlinear regression calculation and kinetic parameter determination.

### Saccharification assays

Saccharification assays were performed in 50 mL Falcon tubes (BD Bioscience) containing 5% (w/v) wheat straw in 50 mM sodium phosphate buffer (pH 4.8) with addition of tetracycline (12.6 mg/mL) and cycloheximide (10 mg/mL). The final reaction volume was 20 mL. Enzymes were added to the basal medium: industrial cocktail GC220 (Genencor-Danisco, Rochester, NY, USA) from *T. reesei *and Novozyme 188 (Novozyme, Franklinton, NC, USA) from *Aspergillus niger, P. cinnabarinus *supernatants containing CDH activity and purified rCDH expressed by *P. pastoris*. *T. reesei *GC220 enzyme cocktail contained 1.41 U CMCase, 0.79 U β-glucosidase, 0.11 U cellobiohydrolase, 3.85 U xylanase, 0.26 U mannanase and 0.14 U pectinase per mg of total protein. *A. niger *Novozyme 188 enzyme cocktail contained 0.06 cellobiohydrolase, 0.18 U CMCase, 1.15 U β-glucosidase, 0.33 U xylanase, 0.20 U mannanase, 0.14 U α-galactosidase and 0.43 U pectinase per mg of total protein. Activities were measured at 37°C, pH 5.0. Saccharification assays were performed in incubators (Infors AG, Switzerland) at 45°C with an orbital shaker (140 rpm) for 96 h. After 96 h of incubation, all the samples were centrifuged at 3500 rpm for 15 min. The supernatants were filtered through a 0.45 μm membrane and carbohydrate was then assayed. Saccharification assays were performed in triplicate.

### Carbohydrate determination

Monosaccharides, cellobiose and gluconic acid generated after hydrolysis of wheat straw were quantified by high-performance anion exchange chromatography (HPAEC) coupled with amperometric detection (PAD) (ICS 3000, Dionex, Sunnyvale, CA, USA) equipped with a Carbo-Pac PA-1 analytical column (250 × 4 mm). Enzymatic reactions were stopped by adding 18 mM NaOH before injection (5 μL) into the HPAEC system. Elution (1 mL/min) was carried out on a sodium acetate gradient (0-250 mM in 25 min). Calibration curves were plotted using galactose, arabinose, glucose, xylose, cellobiose and gluconic acid standards (Sigma-Aldrich), from which response factors were calculated (Chromeleon program, Dionex) and used to estimate the amount of product released in test incubations. All the assays were carried out in triplicate. Reducing sugars released during saccharification assays were quantified by DNS (3, 5-dinitrosalycylic acid) method and visualized at 540 nm as described by Navarro *et al*. [69]. Controls were run with 50 mM acetate buffer pH 5 and references of glucose were ranging from 1 to 10 mM. All assays were performed in triplicate.

## List of abbreviations

CDH: cellobiose dehydrogenase; CMC: carboxymethylcellulose; DCPIP: 2,6-dichlorophenol indophenol; ABTS: 2,2'-azino-bis-(3-ethylbenzthiazoline-6-sulfonic acid); pNP: *para*-nitrophenol; DNS: 3,5-dinitrosalicylic acid; cyt c: cytochrome c; CBM: carbohydrate binding module; rCDH: recombinant cellobiose dehydrogenase.

## Competing interests

The authors declare that they have no competing interests.

## Authors' contributions

MB and JGB designed research. MB and LP carried out experiments. MB and JGB analyzed data. MB, JGB and JCS wrote the paper. All authors read and approved the final manuscript

## References

[B1] Himmel ME Ding SY Johnson DK Adney WS Nimlos MR Brady JW Foust TD Biomass recalcitrance: engineering plants and enzymes for biofuels production Science 2007 315 804 807 10.1126/science.1137016 17289988

[B2] Cherry JR Fidantsef AL Directed evolution of industrial enzymes: An update Curr Opin Biotechnol 2003 14 438 443 10.1016/S0958-1669(03)00099-5 12943855

[B3] Margeot A Hahn-Hagerdal B Edlund M Slade R Monot F New improvements for lignocellulosic ethanol Curr Opin Biotechnol 2009 20 372 380 10.1016/j.copbio.2009.05.009 19502048

[B4] Kersten P Cullen D Extracellular oxidative systems of the lignin-degrading basidiomycete *Phanerochaete chrysosporium* Fungal Genet Biol 2007 44 77 87 10.1016/j.fgb.2006.07.007 16971147

[B5] Martinez D Larrondo LF Putnam N Gelpke MDS Huang K Chapman J Helfenbein KG Ramaiya P Detter JC Larimer F Coutinho PM Henrissat B Berka R Cullen D Rokhsar D Genome sequence of the lignocellulose degrading fungus *Phanerochaete chrysosporium *strain RP78 Nat Biotechnol 2004 22 695 700 10.1038/nbt967 15122302

[B6] Harreither W Sygmund C Dunhofen E Vicuna R Haltrich D Ludwig R Cellobiose dehydrogenase from the ligninolytic basidiomycete *Ceriporiopsis subvermispora* Appl Environ Microbiol 2009 75 2750 2757 10.1128/AEM.02320-08 19270118 PMC2681716

[B7] Li B Nagalla SR Renganathan V Cloning of a cDNA encoding cellobiose dehydrogenase, a hemoflavoenzyme from *Phanerochaete chrysosporium* Appl Environ Microbiol 1996 62 1329 1335 8919793 10.1128/aem.62.4.1329-1335.1996 PMC167898

[B8] Moukha SM Dumonceaux TJ Record E Archibald FS Cloning and analysis of *Pycnoporus cinnabarinus *cellobiose dehydrogenase Gene 1999 234 23 33 10.1016/S0378-1119(99)00189-4 10393235

[B9] Roy BP Dumonceaux T Koukoulas AA Archibald FS Purification and characterization of cellobiose dehydrogenases from the white rot fungus *Trametes versicolor* Appl Environ Microbiol 1996 62 4417 4427 16535462 10.1128/aem.62.12.4417-4427.1996 PMC1389000

[B10] Schmidhalter DR Canevascini G Isolation and characterization of the cellobiose dehydrogenase from the brown-rot fungus *Coniophora puteana *(Schum Ex-Fr) Karst Arch Biochem Biophys 1993 300 559 563 10.1006/abbi.1993.1077 8434937

[B11] Schou C Christensen MH Schulein M Characterization of a cellobiose dehydrogenase from *Humicola insolens* Biochem J 1998 330 565 571 9461557 10.1042/bj3300565 PMC1219174

[B12] Pricelius S Ludwig R Lant NJ Haltrich D Guebitz GM Substrate specificity of *Myriococcum thermophilum *cellobiose dehydrogenase on mono-, oligo-, and polysaccharides related to in situ production of H2O2 Appl Microbiol Biotechnol 2009 85 10.1007/s00253-009-2062-0 19506859

[B13] Zhang R Fan Z Kasuga T Expression of cellobiose dehydrogenase from *Neurospora crassa *in *Pichia pastoris *and its purification and characterization Protein Expr Purif 2010 75 1 63 69 20709172 10.1016/j.pep.2010.08.003

[B14] Hallberg BM Bergfors T Backbro K Pettersson G Henriksson G Divne C A new scaffold for binding haem in the cytochrome domain of the extracellular flavocytochrome cellobiose dehydrogenase Structure 2000 8 79 88 10.1016/S0969-2126(00)00082-4 10673428

[B15] Dumonceaux TJ Bartholomew KA Charles TC Moukha SM Archibald FS Cloning and sequencing of a gene encoding cellobiose dehydrogenase from *Trametes versicolor* Gene 1998 210 211 219 10.1016/S0378-1119(98)00084-5 9573367

[B16] Henriksson G Sild V Szabo IJ Pettersson G Johansson G Substrate specificity of cellobiose dehydrogenase from *Phanerochaete chrysosporium* Biochim Biophys Acta 1998 1383 48 54 10.1016/S0167-4838(97)00180-5 9546045

[B17] Kremer SM Wood PM Production of Fenton reagent by cellobiose oxidase from cellulolytic cultures of *Phanerochaete chrysosporium* Eur J Biochem 1992 208 807 814 10.1111/j.1432-1033.1992.tb17251.x 1396686

[B18] Mason MG Wilson MT Ball A Nicholls P Oxygen reduction by cellobiose oxidoreductase: the role of the haem group FEBS Lett 2002 518 29 32 10.1016/S0014-5793(02)02633-9 11997012

[B19] Samejima M Eriksson KEL A comparison of the catalytic properties of cellobiose: quinone oxidoreductase and cellobiose oxidase from *Phanerochaete chrysosporium* Eur J Biochem 1992 207 103 107 10.1111/j.1432-1033.1992.tb17026.x 1321038

[B20] Henriksson G Johansson G Pettersson G Is cellobiose oxidase from *Phanerochaete chrysosporium *a one-electron reductase? Biochim Biophys Acta 1993 1144 184 190 10.1016/0005-2728(93)90171-B 8369336

[B21] Igarashi K Momohara I Nishino T Samejima M Kinetics of inter-domain electron transfer in flavocytochrome cellobiose dehydrogenase from the white-rot fungus *Phanerochaete chrysosporium* Biochem J 2002 365 521 526 10.1042/BJ20011809 11939907 PMC1222687

[B22] Baminger U Subramaniam SS Renganathan V Haltrich D Purification and characterization of cellobiose dehydrogenase from the plant pathogen *Sclerotium *(Athelia) *rolfsii* Appl Environ Microbiol 2001 67 1766 1774 10.1128/AEM.67.4.1766-1774.2001 11282631 PMC92795

[B23] Dekker RFH Cellobiose dehydrogenase produced by *Monilia *Sp Meth Enzymol 1988 160 454 463

[B24] Nakagame S Furujyo A Sugiura J Purification and characterization of cellobiose dehydrogenase from white-rot basidiomycete *Trametes hirsuta* Biosci Biotechnol Biochem 2006 70 1629 1635 10.1271/bbb.50692 16861797

[B25] Temp U Eggert C Novel interaction between laccase and cellobiose dehydrogenase during pigment synthesis in the white rot fungus *Pycnoporus cinnabarinus* Appl Environ Microbiol 1999 65 389 395 9925558 10.1128/aem.65.2.389-395.1999 PMC91037

[B26] Fang J Liu W Gao PJ Cellobiose dehydrogenase from *Schizophyllum commune*: purification and study of some catalytic, inactivation, and cellulose-binding properties Arch Biochem Biophys 1998 353 37 46 10.1006/abbi.1998.0602 9578598

[B27] Subramaniam SS Nagalla SR Renganathan V Cloning and characterization of a thermostable cellobiose dehydrogenase from *Sporotrichum thermophile* Arch Biochem Biophys 1999 365 223 230 10.1006/abbi.1999.1152 10328816

[B28] Vasil'chenko LG Khromonygina VV Karapetyan KN Vasilenko OV Rabinovich ML Cellobiose dehydrogenase formation by filamentous fungus *Chaetomium *sp. INBI 2-26(-) J Biotechnol 2005 119 44 59 10.1016/j.jbiotec.2005.03.023 15996782

[B29] Saha T Ghosh D Mukherjee S Bose S Mukherjee M Cellobiose dehydrogenase production by the mycelial culture of the mushroom *Termitomyces clypeatus* Process Biochem 2008 43 634 641 10.1016/j.procbio.2008.01.025

[B30] Yoshida M Ohira T Igarashi K Nagasawa H Samejima M Molecular cloning and characterization of a cDNA encoding cellobiose dehydrogenase from the wood-rotting fungus *Grifola frondosa* FEMS Microbiol Lett 2002 217 225 230 10.1111/j.1574-6968.2002.tb11479.x 12480108

[B31] Henriksson G Salumets A Divne C Pettersson G Studies of cellulose binding by cellobiose dehydrogenase and a comparison with cellobiohydrolase 1 Biochem J 1997 324 833 838 9210407 10.1042/bj3240833 PMC1218499

[B32] Harreither W Sygmund C Augustin M Narciso M Rabinovich ML Gorton L Haltrich D Ludwig R Catalytic properties and classification of cellobiose dehydrogenases from Ascomycetes Appl Environ Microbiol 2011 77 1804 1815 10.1128/AEM.02052-10 21216904 PMC3067291

[B33] Henriksson G Johansson G Pettersson G A critical review of cellobiose dehydrogenases Journal of Biotechnology 2000 78 93 113 10.1016/S0168-1656(00)00206-6 10725534

[B34] Westermark U Eriksson KE Purification and properties of cellobiose: quinone oxidoreductase from *Sporotrichum pulverulentum* Acta Chemica Scandinavica Series B-Organic Chemistry and Biochemistry 1975 29 419 424 1154954

[B35] Ander P Mishra C Farrell RL Eriksson KEL Redox reactions in lignin degradation: Interactions between laccase, different peroxidases and cellobiose: quinone oxidoreductase J Biotechnol 1990 13 189 198 10.1016/0168-1656(90)90104-J

[B36] Nutt A Salumets A Henriksson G Sild V Johansson G Conversion of O2 species by cellobiose dehydrogenase (cellobiose oxidase) and glucose oxidase: a comparison Biotechnol Lett 1997 19 379 383 10.1023/A:1018315320696

[B37] Pricelius S Ludwig R Lant NJ Haltrich D Guebitz GM In situ generation of hydrogen peroxide by carbohydrate oxidase and cellobiose dehydrogenase for bleaching purposes J Biotechnol 2011 6 2 224 230 10.1002/biot.201000246 21298807

[B38] Henriksson G Ander P Pettersson B Pettersson G Cellobiose dehydrogenase (cellobiose oxidase) from *Phanerochaete chrysosporium *as a wood degrading enzyme. Studies on cellulose, xylan and synthetic lignin Appl Microbiol Biotechnol 1995 42 790 796 10.1007/BF00171963

[B39] Bao WJ Renganathan V Cellobiose oxidase of *Phanerochaete chrysosporium *enhances crystalline cellulose degradation by cellulases FEBS Lett 1992 302 77 80 10.1016/0014-5793(92)80289-S 1587358

[B40] Langston JA Shaghasi T Abbate E Xu F Vlasenko E Sweeney MD Oxidoreductive cellulose depolymerisation by the enzymes cellobiose dehydrogenase and glycoside hydrolase 61 Appl Environ Microbiol 2011 77 19 7007 7015 10.1128/AEM.05815-11 21821740 PMC3187118

[B41] Sigoillot C Lomascolo A Record E Robert JL Asther M Sigoillot JC Lignocellulolytic and hemicellulolytic system of *Pycnoporus cinnabarinus*: Isolation and characterization of a cellobiose dehydrogenase and a new xylanase Enzyme Microb Technol 2002 31 876 883 10.1016/S0141-0229(02)00208-9

[B42] Zamocky M Hallberg M Ludwig R Divne C Haltrich D Ancestral gene fusion in cellobiose dehydrogenases reflects a specific evolution of GMC oxidoreductases in fungi Gene 2004 338 1 14 10.1016/j.gene.2004.04.025 15302401

[B43] Henriksson G Pettersson G Johansson G Ruiz A Uzcategui E Cellobiose oxidase from *Phanerochaete chrysosporium *can be cleaved by papain into two domains Eur J Biochem 1991 196 101 106 10.1111/j.1432-1033.1991.tb15791.x 2001691

[B44] Eggert C Temp U Eriksson KEL The ligninolytic system of the white rot fungus *Pycnoporus cinnabarinus*: purification and characterization of the laccase Appl Environ Microbiol 1996 62 1151 1158 8919775 10.1128/aem.62.4.1151-1158.1996 PMC167880

[B45] Stentelaire C Lesage-Meessen L Oddou J Bernard O Bastin G Ceccaldi BC Asther M Design of a fungal bioprocess for vanillin production from vanillic acid at scalable level by *Pycnoporus cinnabarinus* J Biosci Bioeng 2000 89 223 230 10.1016/S1389-1723(00)88823-4 16232733

[B46] Eggert C Temp U Dean JFD Eriksson KEL Laccase-mediated formation of the phenoxazinone derivative, cinnabarinic acid FEBS Lett 1995 376 202 206 10.1016/0014-5793(95)01274-9 7498542

[B47] Sigoillot C Record E Belle V Robert JL Levasseur A Punt PJ van den Hondel C Fournel A Sigoillot JC Asther M Natural and recombinant fungal laccases for paper pulp bleaching Appl Microbiol Biotechnol 2004 64 346 352 10.1007/s00253-003-1468-3 14600793

[B48] Ohtakara A Mitsutomi M Uchida Y Purification and enzymatic properties of alpha-galactosidase from *Pycnoporus cinnabarinus* Agri Biol Chem 1984 48 1319 1327 10.1271/bbb1961.48.1319

[B49] Ohtakara A Hayashi N Mitsutomi M Purification and some properties of acid beta-galactosidase from *Pycnoporus cinnabarinus* J Ferment Techno 1981 59 325 328

[B50] Herpoel I Moukha S Lesage-Meessen L Sigoillot JC Asther M Selection of *Pycnoporus cinnabarinus *strains for laccase production FEMS Microbiol Lett 2000 183 301 306 10675601 10.1111/j.1574-6968.2000.tb08975.x

[B51] Gnanamani A Jayaprakashvel M Arulmani M Sadulla S Effect of inducers and culturing processes on laccase synthesis in *Phanerochaete chrysosporium *NCIM 1197 and the constitutive expression of laccase isozymes Enzyme Microb Technol 2006 38 1017 1021 10.1016/j.enzmictec.2006.01.004

[B52] Stapleton PC O'Brien MM O'Callaghan J Dobson ADW Molecular cloning of the cellobiose dehydrogenase gene from *Trametes versicolor *and expression in *Pichia pastoris* Enzyme Microb Technol 2004 34 55 63 10.1016/j.enzmictec.2003.08.006

[B53] Yoshida M Ohira T Igarashi K Nagasawa H Aida K Hallberg BM Divne C Nishino T Samejima M Production and characterization of recombinant *Phanerochaete chrysosporium *cellobiose dehydrogenase in the methylotrophic yeast *Pichia pastoris* Biosci Biotechnol Biochem 2001 65 2050 2057 10.1271/bbb.65.2050 11676020

[B54] Conchie J Gelman AL Levvy GA Inhibition of glycosidases by aldonolactones of corresponding configuration Biochem J 1968 106 135 40 5721453 10.1042/bj1060135 PMC1198478

[B55] Parry NJ Beever DE Owen E Vandenberghe I Van Beeumen J Bhat MK Biochemical characterization and mechanism of action of a thermostable beta-glucosidase purified from *Thermoascus aurantiacus* Biochem J 2001 353 117 127 11115405 PMC1221549

[B56] Beeson WT Iavarone AT Hausmann CD Cate JHD Marletta MA Extracellular aldonolactonase from *Myceliophthora thermophila* Appl Environ Microbiol 2010 77 2 650 656 21075873 10.1128/AEM.01922-10 PMC3020561

[B57] Bruchmann EE Schach H Graf H Role and properties of lactonase in a cellulase system Biotechnol Appl Biochem 1987 9 146 159

[B58] Igarashi K Tani T Kawai R Samejima M Family 3 beta-glucosidase from cellulose-degrading culture of the white-rot fungus *Phanerochaete chrysosporium *is a glucan 1,3-beta-glucosidase J Biosci Bioeng 2003 95 572 576 16233459 10.1016/s1389-1723(03)80164-0

[B59] Yoshida M Igarashi K Kawai R Aida K Samejima M Differential transcription of beta-glucosidase and cellobiose dehydrogenase genes in cellulose degradation by the basidiomycete *Phanerochaete chrysosporium* FEMS Microbiol Lett 2004 235 177 182 15158279 10.1016/j.femsle.2004.04.032

[B60] Holtzapple M Cognata M Shu Y Hendrickson C Inhibition of *Trichoderma reesei *cellulase by sugars and solvents Biotechnol Bioeng 1990 36 275 287 10.1002/bit.260360310 18595079

[B61] Strohdeicher M Schmitz B Bringermeyer S Sahm H Formation and degradation of gluconate by *Zymomonas mobilis* Appl Microbiol Biotechnol 1988 27 378 382

[B62] van Dijken JP van Tuijl A Luttik MAH Middelhoven WJ Pronk JT Novel pathway for alcoholic fermentation of delta-gluconolactone in the yeast *Saccharomyces bulderi* J Bacteriol 2002 184 672 678 10.1128/JB.184.3.672-678.2002 11790736 PMC139522

[B63] Tatum EL Barrat RW Frios N Bonner D Biochemical mutant strains of *Neurospora *produced by physical and chemical treatment Am J Bot 1950 37 38 46 10.2307/2438138

[B64] Couturier M Haon M Coutinho PM Henrissat B Lesage-Meesen L Berrin JG *Podospora anserina *hemicellulases potentiate *Trichoderma reesei *secretome for the saccharification of lignocellulosic biomass Appl Environ Microbiol 2010 77 1 237 246 21037302 10.1128/AEM.01761-10 PMC3019743

[B65] Laemmli UK Cleavage of structural proteins during the assembly of the head of bacteriophage T4 Nature 1970 227 680 5 10.1038/227680a0 5432063

[B66] Bradford MM Rapid and sensitive method for quantitation of microgram quantities of protein utilizing principle of protein-dye binding Anal Biochem 1976 72 248 254 10.1016/0003-2697(76)90527-3 942051

[B67] Tien M Kirk TK Lignin peroxidase of *Phanerochaete chrysosporium* Methods Enzymol 1988 161 238 249

[B68] Paszczynski A Huynh VB Crawford R Enzymatic activities of an extracellular, manganese-dependent peroxidase from *Phanerochaete chrysosporium* FEMS Microbiol Lett 1985 29 37 41 10.1111/j.1574-6968.1985.tb00831.x

[B69] Navarro D Couturier M da Silva GGD Berrin JG Rouau X Asther M Bignon C Automated assay for screening the enzymatic release of reducing sugars from micronized biomass Microb Cell Fact 2010 9 58 10.1186/1475-2859-9-58 20637080 PMC2919459

